# Characterization of *Salmonella* spp. Based on CRISPR PCR and FT-IR Approaches: A Pilot Study in Northern Italy

**DOI:** 10.3390/pathogens15030267

**Published:** 2026-03-02

**Authors:** Clara Tramuta, Irene Floris, Monica Pitti, Giulia Federica Cazzaniga, Miriam Cordovana, Daniela Manila Bianchi

**Affiliations:** 1 Istituto Zooprofilattico Sperimentale del Piemonte, Liguria e Valle d’Aosta, S.C. Sicurezza Alimentare, Via Bologna, 148, 10154 Turin, Italy; clara.tramuta@izsplv.it (C.T.); monica.pitti@izsplv.it (M.P.); giuliafederica.cazzaniga@izsplv.it (G.F.C.); manila.bianchi@izsplv.it (D.M.B.); 2 Istituto Zooprofilattico Sperimentale del Piemonte, Liguria e Valle d’Aosta, Centro di Riferimento per la Tipizzazione delle Salmonelle (CeRTiS), Via Bologna, 148, 10154 Turin, Italy; 3 Bruker Daltonics GmbH & Co. KG, 28359 Bremen, Germany; miriam.cordovana@bruker.com

**Keywords:** characterization, CRISPR PCR, FT-IR, *Salmonella* strains

## Abstract

The aim of the present study was to employ a genotypic method (CRISPR PCR) and a phenotypic method (FT-IR combined with Linear Discriminant Analysis, LDA) for the rapid identification and discrimination of major virulent *Salmonella* serovars. Specifically, a total of 24 *Salmonella* Enteritidis, 24 *Salmonella* Typhimurium, 24 *S.* Typhimurium 4,5,12:i:-, and 14 *Salmonella* Infantis strains, previously serotyped according to the Kaufmann–White scheme, were analyzed. CRISPR PCR generated serotype-specific amplicons: 850 bp for *S.* Enteritidis, 700 bp and 2000 bp for *S.* Typhimurium, 1300 bp and 1500 bp for *S.* Typhimurium 4,5,12:i:-, and 1000 bp and 1900 bp for *S.* Infantis. FT-IR spectra of all serotypes were analyzed by LDA, which revealed a clear separation of three serotype-defined groups: *S.* Enteritidis, *S.* Infantis, and a group comprising *S.* Typhimurium and its monophasic variant 4,5,12:i:-. Further discrimination based on CRISPR-defined classes was observed within each serovar: two classes for *S.* Typhimurium, two for *S.* Typhimurium 4,5,12:i:-, and three for *S.* Infantis. Overall, the combined CRISPR PCR and FT-IR-LDA approach provides preliminary evidence supporting the potential application of a combined approach for the epidemiological surveillance of *Salmonella*.

## 1. Introduction

*Salmonella enterica* is widespread in nature, with over 2600 different serovars, and can infect the intestines of animals and humans, causing salmonellosis [1]. Human salmonellosis is primarily contracted through the consumption of contaminated water, foods of animal origin, and raw or undercooked fruits and vegetables [2]. Transmission can also occur via contact with contaminated surfaces, tools, or food handled by infected individuals [3].

According to the annual report of the European Food Safety Authority (EFSA) and the European Centre for Disease Prevention and Control (ECDC) [4], *Salmonella* enterica is the second most common foodborne pathogen in humans in the European Union [4]. In 2023, 77,486 confirmed cases of human salmonellosis were reported, corresponding to a notification rate of 18.0 cases per 100,000 population. The most frequently isolated serovars in human infections were *S.* Enteritidis (70.8%), *S.* Typhimurium (8.9%), its monophasic variant *S.* 4,5,12:i:- (5.1%), and *S.* Infantis (2%) [4].

In poultry flocks, the National Salmonellosis Control Plan (NCCP 2022–2024) [5] identifies *S.* Enteritidis, *S.* Typhimurium, and the monophasic variant of *S.* Typhimurium as the serovars of greatest epidemiological relevance. Regulation (EU) 2073/2005 [6], as amended, lists these serovars as food safety criteria in fresh poultry meat.

Isolation and typing of *Salmonella* strains along the food chain are therefore essential; however, traditional methods, including the official serotyping method UNI EN ISO 6579-3:2017 [7], are time-consuming, costly, and require specialized personnel. Early identification of these serovars is critical for effective control. To reduce time and costs, the development of innovative, rapid, and easily applicable methods is therefore highly desirable.

The Clustered Regularly Interspaced Short Palindromic Repeats (CRISPR) molecular complex has the function of providing prokaryote cells with a form of acquired immunity, representing a kind of “immune system” capable of eliminating foreign DNA [8]. At the genetic level, the CRISPR complex consists of DNA segments containing short palindromic repeats, separated at regular intervals by small spacer DNA segments [9]. The characterization of CRISPR alleles provides information on the content of spacers to perform typing and subtyping of bacterial strains. In recent years, CRISPR sequences have been identified in about 40% of bacterial genomes, including *Escherichia coli*, *Salmonella* spp., and *Staphylococcus aureus* [10]. *Salmonella* contains two different CRISPR loci (CRISPR 1 and CRISPR 2) related to some of the main *Salmonella* serovars [11,12], such as *S.* Enteritidis and *S.* Typhimurium. Recently, molecular typing techniques have been successfully applied as an alternative or support to traditional methods, as they ensure more accurate characterization, faster execution and application to a wider variety of bacterial species. Size determination of the PCR product can be utilized as a quick screen within the context of an outbreak to rapidly compare and identify isolates with CRISPR arrays that are the same size as that of the outbreak strain [11]. While the fundamental mechanisms of CRISPR systems are well-documented, their application as practical typing tools in regional surveillance programs requires further exploration to evaluate their applicability and cost-effectiveness compared to gold-standard methods.

Fourier transform infrared spectroscopy (FT-IR) is based on the analysis of the biomolecular composition of the intact microbial cells and allows the rapid discrimination of microorganisms at different taxonomic levels [13,14,15]; it can be applied in many fields, including clinical [16,17,18,19,20] and food safety [21,22,23]. Compared with other typing methods, it is a rapid and cost-effective method [23,24].

In microbiology, IR spectroscopy has numerous applications [15] described in the literature: strain typing [11,14,23,25,26,27], discrimination of biofilm-producing strains [28,29], differentiation between living and dead cells [22], detection of stress-injured cells [30] and outbreak investigation [18,20,31].

In the field of food safety, FT-IR spectroscopy has been used to investigate both pathogenic [31] and probiotic bacterial strains [32,33,34,35,36].

FT-IR-based analysis is particularly suitable for *Salmonella* enterica, which has high antigenic diversity associated with clinical relevance [14]. For this reason, numerous scientific studies relating to this bacterial species are available in the bibliography [14,22,23,26,27,28,29,30,37]. FT-IR spectroscopy is often integrated with other advanced techniques, including genetic analysis, to provide more in-depth information on microbial functions [17,27].

The aim of this pilot study was to compare, for the first time, a genotyping approach based on CRISPR sequences with a phenotypic characterization method using FT-IR as diagnostic tools. These approaches, which are faster and simpler than traditional serotyping and more cost-effective than whole-genome sequencing, were evaluated for their ability to differentiate clinically relevant *Salmonella* strains not only at the serovar level but also at the individual strain level. In particular, Linear Discriminant Analysis (LDA) was applied to FT-IR spectra to assess whether infrared bacterial typing (IRBT) captures serovar- and CRISPR-associated biochemical signatures in *Salmonella*, providing a supervised multivariate approach for strain discrimination.

## 2. Materials and Methods

### 2.1. Salmonella Strains

Based on the serovars of greatest epidemiological interest, a total of 86 *Salmonella* strains selected from the collection of the Piedmont Regional Reference Centre for *Salmonella* Typing (Centro di Riferimento per la Tipizzazione delle Salmonelle, CeRTiS) were analyzed. Isolates, collected via laboratory passive surveillance in Piedmont and Liguria regions (Northwest Italy) in the period of 2007–2024, were identified by MALDI TOF/TOF mass spectrometry (Bruker Daltonics GmbH & Co. KG, Bremen, Germany) and successively serotyped according to the Kaufmann–White classification scheme [38]. The following serovars were selected for this study:

(i) *S.* Enteritidis (*n*. 24) of animal, food and environmental origin (Table 1);
(ii) *S.* Typhimurium (*n*. 24) of animal and food origin (Table 2);
(iii) Monophasic variant of *S.* Typhimurium (*S.* 4,5,12:i:-) (*n*. 24) of human origin (Table 3);
(iv) *S.* Infantis (*n*. 14) of human, food and animal origin (Table 4).

### 2.2. DNA Extraction and CRISPR PCR Approach

Genomic DNA from each isolate was extracted by using an Instagene matrix (Bio-Rad, Richmond, CA, USA). The CRISPR 2 primer pair (B1 and B2) used for monoplex PCR were taken from Fabre et al. (2012) [11]. The specificity of the method was checked in silico on CRISPR 2 sequences of representative *Salmonella* strains (e.g., *S.* enteritidis JF725428 and JF72543; *S.* Typhimurium JF725638; *S.* 4,5,12:i:- JF725292 and JF725259) using multiple-alignment software provided in the BioEdit package, version 7.2.6.1. PCR was then performed in a reaction volume of 25 μL using 1X DreamTaq Hot Start PCR Master Mix (Thermo Scientific, Waltham, MA, USA), 10 μM of each primer and 5 μL of DNA. Amplification was carried out in a thermal cycler CFX (Bio-Rad, Richmond, CA, USA) using an initial denaturation for 3 min at 95 °C, 35 cycles consisting of denaturation for 30 sec at 95 °C, annealing for 1 min at 59 °C, and extension for 1 min at 72 °C, followed by a final extension for 10 min at 72 °C. Electrophoresis of amplified products was carried out using 2% agarose gel in 1X TAE running buffer. The agarose gel was then stained with GelRed (Biotium, Fremont, CA, USA) and visualized by ultraviolet transillumination Gel-Doc (Bio-Rad, Richmond, CA, USA).

### 2.3. FT-IR Approach

The analyzed strains were incubated at 37 °C for 24 h on Blood Agar under standard atmospheric conditions. Sample preparation was performed using Biotyper IR Kits (Bruker Daltonics GmbH & Co. KG, Bremen, Germany) following the manufacturer’s instructions. Briefly, a bacterial suspension was prepared by collecting several colonies from the confluence zone of the culture plate and resuspending them in 50 μL of 70% ethanol. The suspension was vortexed, and 50 μL of deionized water was added. After mixing by pipetting, 15 μL of the suspension was placed in five technical replicates on a 96-spot silicon IRBT target plate.

The quality control standards, Infrared Test Standards (IRTS 1 and IRTS 2) provided with the kit, were resuspended in 90 μL of deionized water and 90 μL of absolute ethanol, then vortexed. We applied 12 μL of each suspension in duplicate to the plate. The plate was dried for 15–20 min at 35 °C.

Spectra were acquired in transmission mode in the spectral range of 500–4000 cm^−1^ (mid IR) using OPUS V7.5.18 (Bruker Optics, GmbH, Ettlingen, Germany) and IR Biotyper Client Software V3.0 (Bruker Daltonics GmbH, Bremen, Germany), applying the manufacturer’s recommended settings. In the IR Biotyper system, after acquisition, the spectra are smoothed using the Savitzky–Golay algorithm, the second derivative over 9 datapoints is calculated, and the spectra are then vector-normalized, to correct variations related to spectra acquisition and sample amount. No outlier spectra were actually found, and all the recorded spectra were included in the dataset.

Spectral analysis was performed using Principal Component Analysis (PCA) and Linear Discriminant Analysis (LDA), with serovar (*S.* Enteritidis, *S.* Typhimurium, *S.* 4,5,12:i:-, and *S.* Infantis) as the class label, to evaluate the discriminatory ability of IRBT spectra. The selected spectral regions (700–1300, 1400–1500, and 2800–3000 cm^−1^) were chosen based on preliminary exploratory analysis, which identified these intervals as those exhibiting the highest inter-group variability. These regions correspond to well-known biochemical vibrational bands, including nucleic acids, proteins, lipids, and CH stretching modes, which are commonly reported in FTIR-based discrimination studies. Subsequently, each serovar (*S.* Typhimurium, monophasic variant of *S.* Typhimurium, and *S.* Infantis) was analyzed separately by PCA/LDA using the same spectral regions. The number of LDA components was selected based on the deviation plot and by retaining those maximizing class separation while minimizing within-class variance, in accordance with standard discriminant analysis procedures. Within each serovar, strains were assigned to discrete classes based on their CRISPR amplicon patterns, which were used as the grouping variable. For *S.* enteritidis, spectral distances were calculated using the Euclidean distance metric, and hierarchical clustering was performed using the UPGMA algorithm (average linkage). Clusters were defined using a cut-off value of 0.203, automatically calculated by the software on the bases of Simpson’s diversity index and mean coherence.

## 3. Results

### 3.1. CRISPR PCR Approach

**Figure 1:**
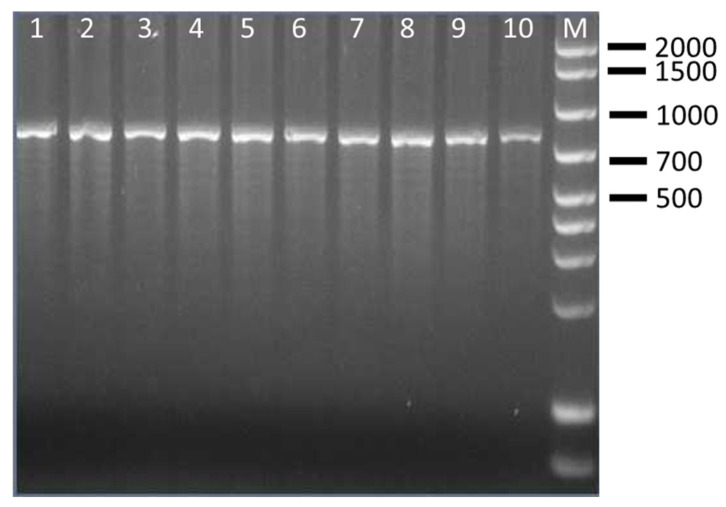
Amplification products obtained by CRISP 2 PCR on representative *S.* Enteritidis strains. Lanes 1–4: ID strains 1–4; lanes 5–7: ID strains 6–8; Lines 9–10: ID strains 13–14; M: 100–base pair DNA ladder plus.

**Figure 2:**
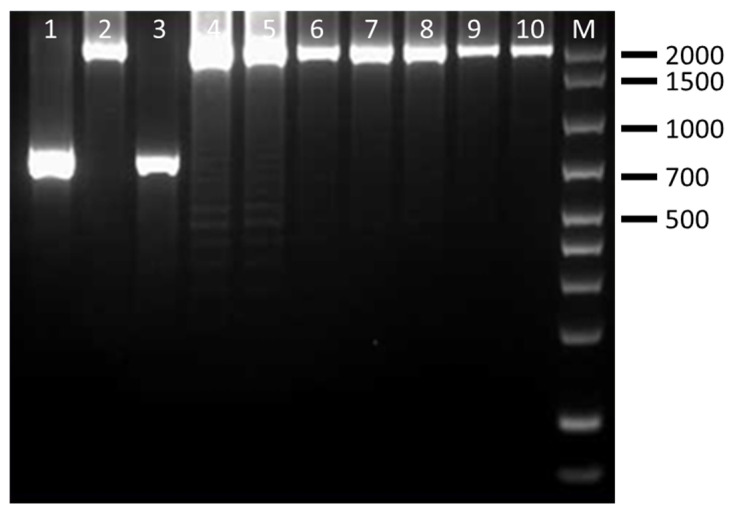
Amplification products obtained by CRISP 2 PCR on representative *S.* Typhimurium strains. Lanes 1–2: ID strains 4–5; lanes 3–6: ID strains 13–16; Lines 7–10: ID strains 16–19; M: 100–base pair DNA ladder plus.

**Figure 3:**
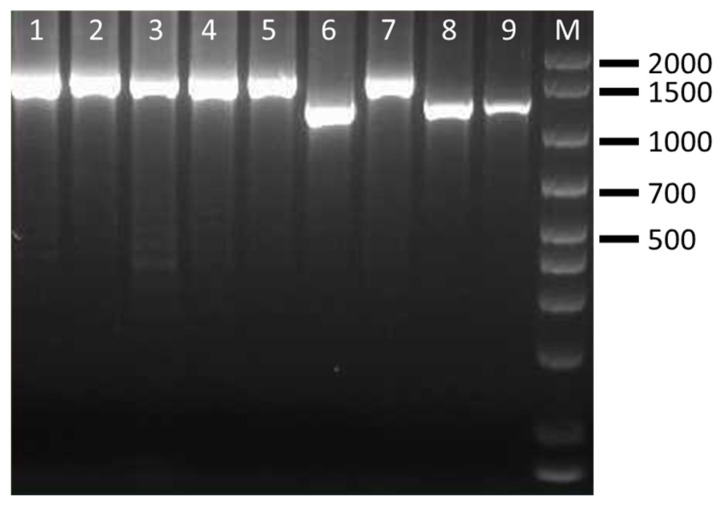
Amplification products obtained by CRISP 2 PCR on representative *S.* 4,5,12:i:- strains. Lanes 1–3: ID strains 2–4; lanes 4–6: ID strains 5–7; Lines 7–9: ID strains 11–13; M: 100–base pair DNA ladder plus.

**Figure 4:**
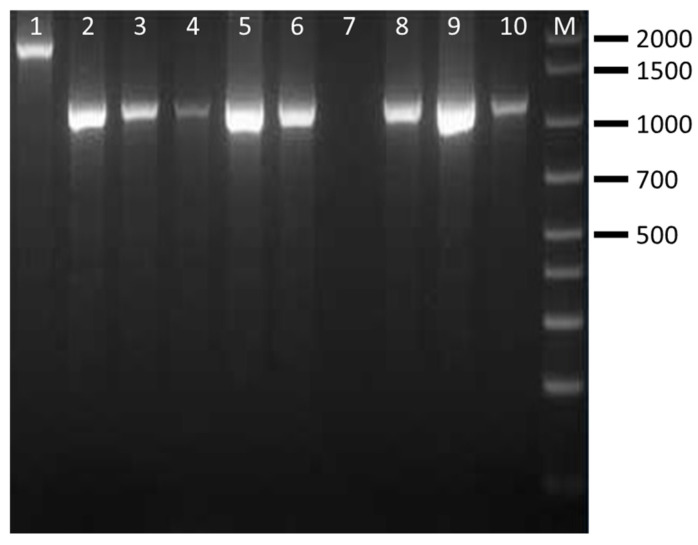
Amplification products obtained by CRISP 2 PCR on representative *S.* Infantis strains. Lanes 1–3: ID strains 5–7; lanes 4–6: ID strains 8–10; Lines 7–10: ID strains 11–14; M: 100–base pair DNA ladder plus.

The CRISPR PCR used in this study was able to discriminate four clinically relevant serovars of *Salmonella* spp. The results showed that CRISP 2 monoplex PCR generated *Salmonella* serovar-specific amplicons of ~850 bp for *S.* Enteritidis (100% of strains), ~700 bp/2000 bp for *S.* Typhimurium (100% of strains), ~1300 bp/1500 bp for *S.* Typhimurium 4,5,12:i:- (100% of strains) and ~1000 bp/1900 bp for *S.* Infantis (85.7% of strains) (Table 5). Separation of the fragments by agarose gel electrophoresis was clear, easy to interpret and showed no interaction among *Salmonella* species. Amplificons obtained from representative *Salmonella* strains are shown in Figure 1, Figure 2, Figure 3 and Figure 4 and are comparable to those obtained previously [11,12].

### 3.2. FT-IR Approach

A total of 430 spectra (120 *S.* Enteritidis, 120 S. Typhimurium, 120 monophasic variants of *S.* Typhimurium, and 70 *S.* Infantis) were acquired.

**Figure 5:**
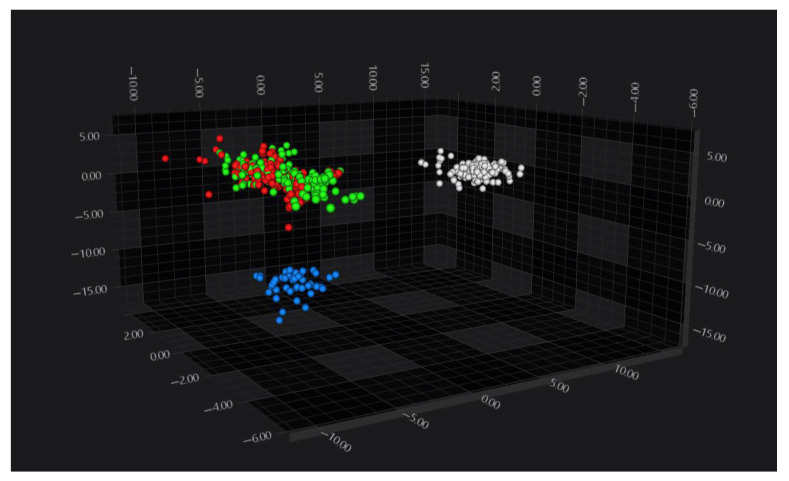
Three-dimensional scatter plot of the LDA scores showing the separation of *Salmonella* serovars in the IR spectral space: *S. Enteritidis* (white), *S. Infantis* (blue), *S. Typhimurium* (red), and its monophasic variant (green).

FT-IR spectra from all serovars were analyzed by PCA/LDA, which resulted in a clear separation of three serovar-defined groups: *S.* Enteritidis, *S.* Infantis, and a group comprising *S.* Typhimurium and its monophasic variant (Figure 5).

**Figure 6:**
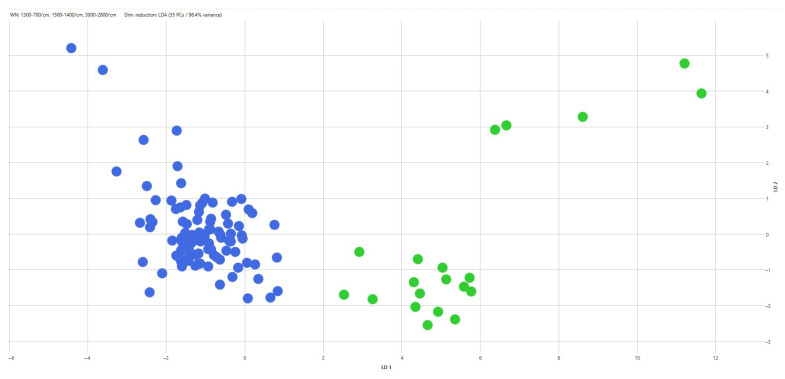
Two-dimensional scatter plot of the LDA scores for *S. Typhimurium*, showing the separation of CRISPR-defined classes: class A (blue) and class B (green).

**Figure 7:**
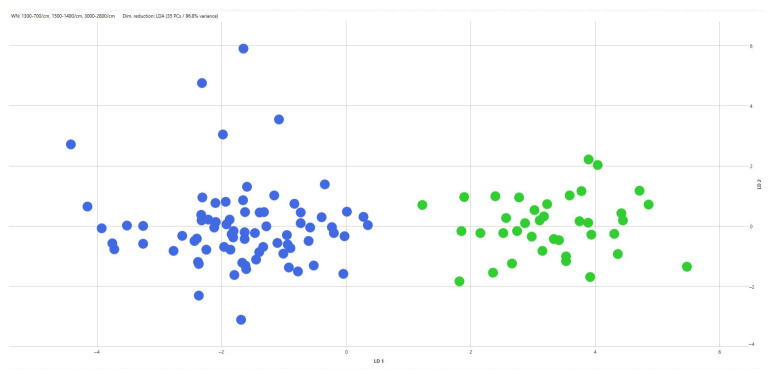
Scatter plot of the LDA scores for the monophasic variant of *S. Typhimurium*, showing the separation of CRISPR-defined classes: class A (blue) and class B (green).

**Figure 8:**
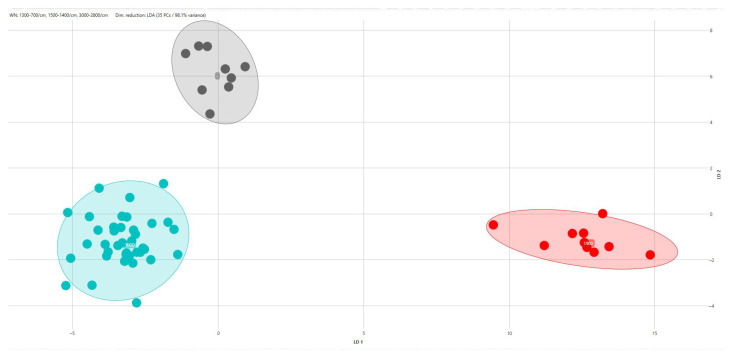
Scatter plot of the LDA scores for *S. Infantis*, showing the separation of CRISPR-defined classes: class A (red), class B (light blue), and class C (gray).

**Figure 9:**
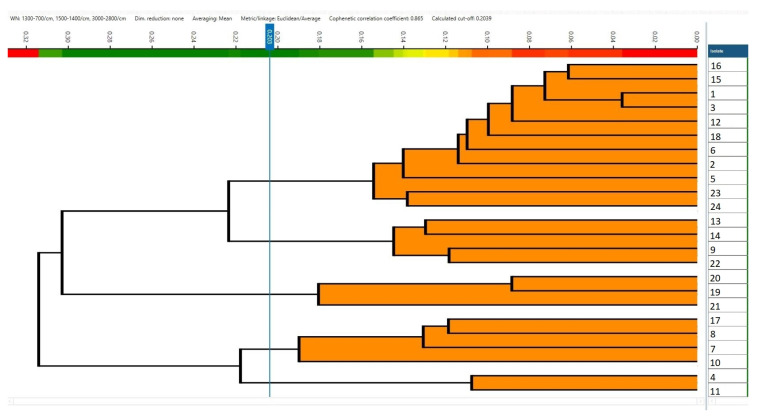
Hierarchical clustering of *S.* enteritidis isolates based on FT-IR spectra obtained using the IR Biotyper system.

When PCA/LDA was applied separately within each serovar using CRISPR amplicon profiles as predefined classes, additional discrimination among CRISPR genotypes was observed (Table 5). Within *S.* Typhimurium, two CRISPR-defined classes were discriminated (Figure 6), corresponding to strains carrying a 2000 bp amplicon and those carrying a 700 bp amplicon. Similarly, two CRISPR-defined classes were discriminated within the monophasic variant of *S.* Typhimurium (Figure 7), associated with 1500 bp and 1300 bp amplicons, respectively. Within *S.* Infantis, three CRISPR-defined groups were separated by LDA, corresponding to strains with a 1900 bp amplicon, those with a 1000 bp amplicon, and strains lacking detectable amplicons (Figure 8). For *S.* enteritidis, hierarchical clustering of FT-IR spectra identified five distinct clusters among the *S.* enteritidis isolates using a cut-off value of 0.203 (average Euclidean distance). The dendrogram showed a high cophenetic correlation coefficient (CCC = 0.865), indicating a good representation of the original spectral distances. No association was observed between the origin of the isolates and cluster formation (Figure 9).

## 4. Discussion

In Italy, Salmonellosis is the most common detected food-borne infection, with over 3000 reported cases annually [4]. Based on the distribution of serovars collected by CeRTiS, the most common serovars detected in 2024 in the Piedmont and Liguria regions were *S.* 4, [5],12:i:- (46.94%), *S.* Typhimurium (11.13%), and *S.* Enteritidis (8.90%) [39,40]. Prevention, control, and monitoring programs to reduce the presence of *Salmonella* strains in the food chain, together with a better knowledge of circulating *Salmonella* serovars and contamination sources, may contribute to defining food safety intervention priorities and implementing appropriate control measures [41].

Approaches alternative to serotyping are useful to rapidly identify and discriminate the most relevant *Salmonella* serovars. For these reasons, the aim of the present study was to increase the capacity for analysis and characterization of *Salmonella* strains using valid, rapid, inexpensive and not extremely laborious methods that can potentially be applied in molecular biology and microbiology laboratories in order to improve the surveillance of *Salmonella* infections. In general, the application of the methods used in this pilot study, on locally circulating strains of human, animal and food origin, makes it possible to define the genotypic and phenotypic profiles of bacterial strains to set up epidemiological investigations with the identification of public health-relevant profiles and to improve the ability to monitor the emergence of particular epidemic clones. The results showed a correspondence between CRISPR amplicon profiles and FT-IR biochemical signatures. However, it is important to note that this relationship remains an observed correlation within the current sample set rather than a demonstrated mechanistic link. While both methods appear to reflect serovar-specific characteristics, these findings could also be influenced by the clonal population structure of the isolates. Therefore, further genomic and biochemical studies are required to confirm whether specific CRISPR genotypes directly influence the phenotypic expression levels captured by FT-IR spectroscopy.

*Salmonella* contains two different CRISPR loci that correlate with some of the main *Salmonella* serovars, such as *S.* Enteritidis and *S.* Typhimurium, considered the most common human serovars as well as the most relevant for public and veterinary health. CRISPR polymorphism has been optimized and applied for the identification and characterization of clinically relevant *Salmonella* serovars in recent years [11,12,42,43,44]. The CRISPR-PCR method, based on the CRISPR 2 locus here applied, made possible in a single analytical run and using a single pair of primers, can discriminate *Salmonella* strains belonging to four different serovars, generating specific amplicons for *S.* Enteritidis, *S.* Typhimurium, the monophasic variant of *S.* Typhimurium (*S.* 4,5,12:i:-) and *S.* Infantis. The data obtained show that this approach can be used by official and microbiology laboratories both as a new method for identifying clinically relevant *Salmonella* serovars, and as a method of support for the classification of serovars according to the White–Kauffmann and Le Minor scheme. In addition, the method allows us to simplify the typing of *Salmonella* by multiplex PCR of *flj*B, *gyr*B and *yef*Q genes, used today as a standard method to discriminate *S.* Typhimurium from its monophasic variant 4,5,12:i:-. While the CRISPR-PCR assay demonstrates potential for rapid screening, comprehensive metrics for sensitivity and specificity remain to be established through broader validation studies.

*Salmonella enterica* exhibits high antigenic diversity, which is closely associated with its clinical relevance [26]. Consequently, numerous studies have applied FT-IR-based strain typing for the characterization of *Salmonella* isolates [14,22,23,30]. However, FT-IR spectra alone may not provide comprehensive insights into microbial genotypes and functions, and they are best integrated with advanced molecular approaches, such as CRISPR analysis, to achieve more detailed strain characterization [17,27].

In this study, LDA was applied to FT-IR spectra to assess the separation of predefined serovar- and CRISPR-defined classes. The LDA results suggest that differences in serovar and CRISPR profiles are accompanied by consistent changes in the biochemical composition of bacterial cells, as captured by IRBT.

Our preliminary results indicate that FT-IR, combined with LDA, can discriminate major serovars, including *S.* Enteritidis, *S.* Infantis, and *S.* Typhimurium. However, FT-IR could not fully separate *S.* Typhimurium from its monophasic variant 4,5,12:i:-, highlighting the need for complementary molecular approaches. In such cases, CRISPR-PCR remains an essential complementary tool for achieving full serovar discrimination. This study was designed as an exploratory proof-of-concept investigation with a limited sample size. Consequently, a formal independent training/testing split was not performed. This limitation should be considered when interpreting the generalizability of the proposed FT-IR model. Additional studies with larger sample sizes are required to further evaluate the discriminatory performance of FT-IR, particularly for these closely related serovars. Using a cut-off of 0.203, five spectral clusters were identified for *S.* Enteritidis among the analyzed *S.* Enteritidis isolates. While these clusters suggest the presence of five distinct groups, genomic data would be required to confirm their biological significance. In the case of S. Enteritidis, hierarchical clustering revealed multiple spectral clusters; however, no clear association with isolate origin was observed. We acknowledge that this indicates that FTIR-based clustering reflects biochemical variability rather than epidemiological or phylogenetic relationships.

Despite the limited dataset of 86 isolates, our findings suggest that this methodology could serve as a valuable screening tool. However, further studies with a larger and more geographically diverse panel are required to confirm its generalizability. This analytical approach could be used to support the official ISO method or as a screening tool for the rapid identification of these relevant serotypes. Moreover, when compared to current gold-standard techniques such as Whole-Genome Sequencing (WGS) or Matrix-Assisted Laser Desorption/Ionization Time-of-Flight (MALDI-TOF), the proposed CRISPR/FT-IR approach offers significant advantages in terms of speed and lower operational costs. However, it lacks the high-resolution genomic depth of WGS and requires rigorous local database calibration to overcome the inherent sensitivity of FT-IR to varying growth conditions.

In conclusion, the observed correlation between CRISPR amplicon profiles and FT-IR biochemical signatures suggests a potential link between genotypic and phenotypic traits in the studied isolates. However, this remains a preliminary observation, and further studies are needed to determine whether this reflects a direct mechanistic relationship or is a consequence of the clonal population structure.

## 5. Conclusions

The method of CRISPR PCR here employed was found to be economical and quick to perform, suggesting it could serve as a promising tool for routine laboratory screening, provided further large-scale validation is conducted. The ‘serovar specificity’ of the observed amplicons requires further confirmation through amplicon sequencing and testing against a broader, more diverse panel of *Salmonella* lineages. By maximizing the separation between predefined biological groups, LDA demonstrated that both serovars and CRISPR genotypes are associated with distinct infrared spectral fingerprints, indicating that genomic variation is reflected in measurable biochemical differences. We therefore consider that it is important to continue this type of investigation in order to evaluate a possible application of the method to other clinically relevant and circulating *Salmonella* serovars.

## Figures and Tables

**Table 1:** Origins of *S.* Enteritidis strains (*n*. 24) characterized.

ID Strain	Origin
1	Animal
2	Food
3	Animal
4	Animal
5	Animal
6	Environmental
7	Food
8	Animal
9	Food
10	Food
11	Food
12	Food
13	Food
14	Animal
15	Environmental
16	Animal
17	Animal
18	Animal
19	Animal
20	Animal
21	Animal
22	Animal
23	Animal
24	Animal

**Table 2:** Origins of *S.* Typhimurium strains (*n*. 24) characterized.

ID Strain	Origin
1	Food
2	Food
3	Food
4	Food
5	Animal
6	Food
7	Food
8	Food
9	Food
10	Food
11	Food
12	Animal
13	Animal
14	Animal
15	Animal
16	Animal
17	Animal
18	Animal
19	Food
20	Animal
21	Animal
22	Food
23	Animal
24	Animal

**Table 3:** Origins of *S.* Typhimurium 4,5,12:i:- strains (*n*. 24) characterized.

ID Strain	Origin
1	Human
2	Human
3	Human
4	Human
5	Human
6	Human
7	Human
8	Human
9	Human
10	Human
11	Human
12	Human
13	Human
14	Human
15	Human
16	Human
17	Human
18	Human
19	Human
20	Human
21	Human
22	Human
23	Human
24	Human

**Table 4:** Origins of *S.* Infantis strains (*n*. 14) characterized.

ID Strain	Origin
1	Human
2	Human
3	Human
4	Human
5	Human
6	Food
7	Food
8	Human
9	Food
10	Animal
11	Animal
12	Animal
13	Animal
14	Animal

**Table 5:** Comparison between *Salmonella* serovar amplicons generated by CRISPR PCR and the separation of corresponding CRISPR-defined classes in FT-IR spectra, as assessed by PCA/LDA.

ID Strain	*S.* Enteritidis		*S.* Typhimurium		*S.* 4,5,12:i:-		*S.* Infantis	
	CRISPR PCR (bp)	FT IR Clusters	CRISPR PCR (bp)	FT IR Classes	CRISPR PCR (bp)	FT IR Classes	CRISPR PCR (bp)	FT IR Classes
1	850	A	2000	A	1500	A	1900	A
2	850	A	2000	A	1500	A	no amplicon	C
3	850	A	2000	A	1500	A	1900	A
4	850	E	700	B	1500	A	1000	B
5	850	A	2000	A	1500	A	1900	A
6	850	A	2000	A	1500	A	1000	B
7	850	D	2000	A	1300	B	1000	B
8	850	D	2000	A	1500	A	1000	B
9	850	B	2000	A	1300	B	1000	B
10	850	D	2000	A	1300	B	1000	B
11	850	E	2000	A	1500	A	no amplicon	C
12	850	A	2000	A	1300	B	1000	B
13	850	B	700	B	1300	B	1000	B
14	850	B	2000	A	1300	B	1000	B
15	850	A	2000	A	1300	B		
16	850	A	2000	A	1300	B		
17	850	D	2000	A	1500	A		
18	850	A	2000	A	1500	A		
19	850	C	2000	A	1500	A		
20	850	C	700	B	1500	A		
21	850	C	700	B	1500	A		
22	850	B	2000	A	1500	A		
23	850	A	2000	A	1500	A		
24	850	A	2000	A	1500	A		

## Data Availability

Data is contained within the article.
